# Mass drug administration and the sustainable control of schistosomiasis: an evaluation of treatment compliance in the rural Philippines

**DOI:** 10.1186/s13071-018-3022-2

**Published:** 2018-07-31

**Authors:** Marianette T. Inobaya, Thao N. Chau, Shu-Kay Ng, Colin MacDougall, Remigio M. Olveda, Veronica L. Tallo, Jhoys M. Landicho, Carol M. Malacad, Mila F. Aligato, Jerric B. Guevarra, Allen G. Ross

**Affiliations:** 1 Menzies Health Institute Queensland, Griffith University, Gold Coast, Queensland Australia; 2 Department of Health, Research Institute for Tropical Medicine, Muntinlupa City, Philippines; 3 Discipline of Public Health, School of Health Sciences, Flinders University, Adelaide, Australia

**Keywords:** Schistosomiasis, Mass drug administration (MDA), Compliance, Philippines

## Abstract

**Background:**

Preventive chemotherapy is the current global control strategy for schistosomiasis. The WHO target coverage rate is at least 75% for school-aged children. In the Philippines, the reported national coverage rate (43.5%) is far below the WHO target. This study examined the factors associated with non-compliance to mass drug administration.

**Methods:**

A cross-sectional survey was conducted in 2015 among 2189 adults in the province of Northern Samar, the Philippines using a structured face-to-face survey questionnaire.

**Results:**

The overall rate of non-compliance to mass drug administration (MDA) in the last treatment round was 27%. Females (aOR = 1.67, *P* = 0.033) were more likely to be non-compliant. Respondents who believed that schistosomiasis was acquired by open defecation and poor sanitation (aOR = 1.41, *P* = 0.015), and by drinking unclean water (aOR = 2.09, *P* = 0.001) were more likely to refuse treatment. Uncertainties on whether schistosomiasis can be treated (aOR = 2.39, *P* = 0.033), their fear of adverse reactions to praziquantel (aOR = 1.94, *P* = 0.021), misconceptions about alternative forms of treatment (aOR = 1.45, *P* = 0.037), and that praziquantel is used for purposes other than deworming (aOR = 2.15, *P* = 0.021) were all associated with a higher odd of non-compliance. In contrary, being a farmer (aOR = 0.62, *P* =0.038), participation in past MDA (aOR = 0.30, *P* < 0.001), informed about impending MDA (aOR = 0.08, *P* < 0.001), and having heard of schistosomiasis (aOR = 0.22, *P* = 0.045) were all significantly associated with reduced non-compliance.

**Conclusions:**

To improve drug compliance for schistosomiasis there is an urgent need for intensive health education campaigns before conducting MDA that would not only provide disease specific information, but also deal with prevailing misconceptions about transmission, prevention, treatment, and drug side-effects.

## Background

Schistosomiasis is infection with trematodes of the genus *Schistosoma* with five species known to infect humans*.* It is endemic in 78 countries, 42 of which are in the Africa, 16 in the Eastern Mediterranean, 10 in the Americas, six in the Western Pacific, three in Southeast Asia and one in Europe [1]. In 2016, the disability-adjusted life years (DALYs) due to schistosomiasis was estimated at 2.521 million and WHO estimated that 111.2 million school-aged children and 92.5 million adults are in need of preventive chemotherapy for schistosomiasis [2, 3].

Preventive chemotherapy, through regular mass drug administration (MDA) of praziquantel, was endorsed by World Health Assembly in 2001 as the main strategy for schistosomiasis control through WHA resolution 54.19. Treatment with praziquantel at a dose of 40 mg/kg body weight aims to reduce morbidity and mortality, and prevent new infection by limiting transmission through the reduction of the human reservoir [4]. The WHO target is to regularly treat a minimum of 75% and up to 100% of all school-age children at risk of morbidity by 2010 [5]. This was not achieved and a new set of goals for 2020 was declared which were 100% geographic coverage, 75% national coverage and <5% prevalence of heavy infections [1].

In the Philippines, zoonotic *Schistosoma japonicum* infection is endemic in 28 provinces in 12 regions of the country, with an estimated 28 million people at risk of infection. In the national prevalence survey conducted from 2005–2008, the estimated mean human prevalence was 1.30% (range 0.08–6.30%) [6]. However, an epidemiological survey conducted 2012 in the province of Northern Samar on over 18,000 residents from 22 endemic villages found that the prevalence ranged between 5–48% [7]. For over two decades human MDA has been the cornerstone of schistosomiasis control in the country. In an effort to eliminate schistosomiasis as a public health problem, and to protect the exposed population from developing chronic infection, the Department of Health (DOH) has implemented the conduct of annual MDA in 24 endemic provinces among those aged 5–65 years commencing in 2009. The aim was to attain at least 85% drug coverage for at least three years or until disease elimination (human prevalence < 1%) was achieved [8]. From a prevalence of > 10% in 1990, it was reduced to less than 5% after 1995. However, the goal of disease elimination remains elusive as prevalence rates in 2013–2015 reported in 10 out of 13 provinces remained above 1% [9].

The success of MDA in reducing the human prevalence of infection, and preventing transmission depends on the treatment coverage rate, the frequency of treatment campaigns, and compliance to treatment [10]. Coverage rate is often defined in studies as the percentage of the targeted population who received the drug, while compliance is the percentage who received and swallowed the drug [11]. The most recent global coverage rate reported by WHO in 2016 was found to be 35.6%; 53.0% among school-aged children and 14.3% among adults [3]. Global coverage rates for schistosomiasis have consistently been lower than for the other neglected tropical diseases (e.g. lymphatic filariasis, onchocerciasis and soil-transmitted helminthiases) [12]. In the WHO strategic plan (2012–2020) for schistosomiasis control, one of the milestones set forth was to achieve at least 75% coverage among school-aged children in 50% or more of the countries requiring preventive chemotherapy by 2015 [1]. A review of WHO’s Preventive Chemotherapy Databank showed that in 2015, only eight out of the 36 countries have reported coverage rates greater than 75% [13].

In addition to the problem of low coverage rates is the existence of a gap between the coverage reported by government or international agencies and the actual proportion of the population who consumed the treatment [14, 15]. A review of MDA programmes for lymphatic filariasis in India revealed a difference of 22% between the average reported coverage and compliance rates [16]. In Zanzibar, community-wide treatment of praziquantel among adults had a coverage rate of 60–71% while the compliance was lower at 50–60% [17]. Challenges in attaining high compliance rates include religious beliefs, poor acceptability of the tablets due to its size and quantity, fear of treatment side-effects, and inadequate health education [15, 18–20].

Included in the countries with below target coverage rates in 2015 was the Philippines at 43.5%, the highest rate since the 2009 DOH order to implement MDA, but only halfway to the country’s own ambitious target of 85% [13]. Recent studies in the provinces of Samar and Leyte reported coverage rates of 60% and 48%, respectively [21, 22]. There has been no previous published report on patient compliance for free schistosomiasis treatment in the country. Given that MDA is the primary strategy for schistosomiasis control in the country, it is imperative to identify MDA non-compliers so that targeted strategies to improve compliance can be implemented. Compliance to schistosomiasis MDA and the factors associated with non-compliance have not been as widely studied in the country, or even globally. The aim of this study was to determine the socio-demographic characteristics, knowledge, and attitudes associated with drug non-compliance.

## Methods

### Study design and study area

A cross-sectional schistosomiasis survey on knowledge and attitudes, as well as MDA compliance was conducted in 2015 among residents 18 years of age and older in the endemic municipalities of Laoang and Palapag in Northern Samar, the Philippines in January to March 2015. A recent (2012) parasitological and demographic survey in the study area covered 18,221 people ages 5–65 years old. The surveyed population had a mean age of 28.5 years (95% CI: 25.9–27.1), and 51% were male [23]. This survey revealed an overall human prevalence of 27% [23]. Mass drug administration has been the primary control strategy since being implemented by the DOH in 2000 [8]. The most recent MDA was conducted in the area in October 2014 to January 2015. Northern Samar belongs to a region known to have a high level of schistosomiasis endemicity, with more than 50% of the population residing within endemic areas. Family incomes are far below the national mean income with most head of households engaged in rice farming. Aside from schistosomiasis, soil-transmitted helminths, acute respiratory infections, diarrheal diseases, and other communicable diseases are highly prevalent [23].

### Study procedures

A cross-sectional demographic and parasitological survey was conducted in 2012 in 22 villages in Northern Samar. In 2015, individuals aged 18 years-old and above were randomly selected from these villages, with number of respondents selected per barangay (village) proportional to the size of the village population, using the previous survey’s surveyed population as the sampling frame for the present study [23]. The computed minimum sample size required to attain a power of 80% to detect an odds ratio of 2.0–2.2 was 1936. Eighteen years is the age of consent in the Philippines. Hence, they had the capacity to decide to participate in the survey, and more importantly, in the mass treatment. In 2012, a prevalence survey conducted in the area also showed high prevalence of schistosomiasis among adults, especially in the ages 35–49 years [23].

Local interviewers were trained prior to the start of the survey. The training covered an overview of the protocol of the project, how to invite study participants, obtain informed consent, administer the interview questionnaire, and practice interview sessions. Baseline socio-demographic and the schistosomiasis stool test results were obtained from the 2012 databank. The questionnaire used in the survey had sections on knowledge and attitudes with respect to schistosomiasis and MDA, sources of information, access to health facilities, as well as MDA compliance, delivery, timing notification, and adverse reactions. This structured questionnaire was translated to the local dialect, Waray, and back-translated to English. Adjustments were made on the questions that needed corrections based on the result of the translation and back-translation.

### Data management and analysis

All questionnaires were reviewed by field supervisors before being double-encoded into a customized Microsoft Office Access 2007 data entry system. Cross-checking and analysis of data were performed using STATA SE version 13.1 software (StataCorp LP, College Station, TX, USA). MDA coverage rate was computed as the number who reported that they have participated in the last MDA divided by the total number of respondents who answered this question. Compliance rate, on the other hand, was the number of respondents who consumed all the drugs they received during MDA divided by the denominator used in the coverage rate. Chi-square test was used to explore univariate association of the respondents’ profile, knowledge, and attitudes to schistosomiasis and the likelihood of non-compliance to MDA. Interaction effects between the demographic and knowledge variables were also assessed using Chi-square analysis. All significant (*P* ≤ 0.05) independent variables and interactions were entered into the mixed-effect logistic regression analyses to determine the factors with significant association with non-compliance to MDA. Random barangay and household effects were included in the model to account for the correlation among observations within barangays and households, respectively. Log-likelihood for all levels of both random effects in the mixed models were computed using adaptive Gaussian quadrature with seven integration points. Stepwise backwards regression was used to eliminate factors that were not significantly associated with non-compliance, with a cut-off for statistical significance of *P* ≤ 0.05.

Respondents were classified as MDA compliant if they had participated in the last MDA campaign and consumed all the drugs they received based on self-report. To reduce the possible bias in reporting compliance, measures like assuring confidentiality on answers and no right or wrong answers, and asking the question without influencing the respondent were taken to decrease the possibility of receiving socially-desirable responses like compliance [24]. MDA non-compliant respondents are those who were absent during the drug distribution, present but did not receive the treatment, or did not consume all the drugs received. Participation in previous MDA campaigns refers to whether the respondent received treatment in at least one of the past three MDA campaigns. Wealth status was categorized as ‘wealthy’ if their house flooring is made of cement, tiles or marble, and had galvanized iron roofing and cemented walls. Respondents are classified as ‘poor’ if their house had a soil floor and nipa roof (palm), and the walls were not cemented. All other respondents were classified under the ‘moderate’ wealth status category [25]. The individual history of recent schistosomiasis infection was based on the 2012 stool survey.

## Results

**Fig. 1:**
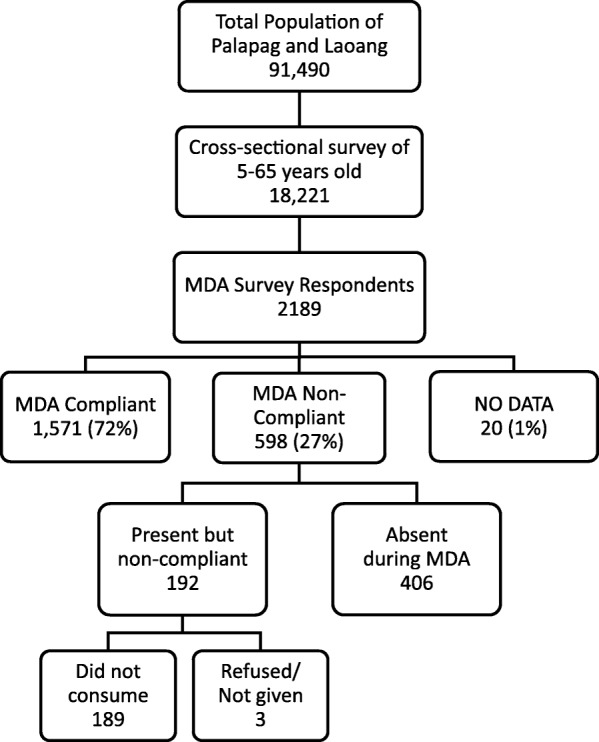
Mass drug administrative (MDA) survey population from the municipalities of Palapag and Laoang, northern Samar, the Philippines

A total of 2189 residents (mean age 42.8 ±12.8 years; 52% female) from 1346 households were interviewed (Table 1), while there were 25 who refused to participate in the survey. Twenty respondents (1%) were either away at the time of treatment or data were missing for classification (Fig. 1). The overall drug coverage rate was 81% (95% CI: 79.4–82.8%) and MDA compliance was 72.4% (95% CI: 70.5–74.3%). There was significant variation in the predicted barangay-specific and household-specific random effects for non-compliance to MDA as indicated by a barangay variance of 0.22 (95% CI: 0.08–0.56) and the household variance of 0.76 (95% CI: 0.32–1.77). Only 8% of the residents had attended college or post-graduate school, 27% were farmers, and only 4% were involved in fishing. In terms of economic status, 61% were considered to be of medium wealth, 92% resided in their own home, 63% owned the land their house was built on, and 38% came from families who owned farms.

**Table 1:** Profile of mass drug administration (MDA) survey respondents from Northern Samar, the Philippines (*n* = 2189)

Profile	*n*	%
Age (years)		
18–30	455	20.8
31–40	569	26.0
41–50	534	24.4
> 50	631	28.8
Mean	42.8	
SD	12.8	
Sex		
Male	1051	48.0
Female	1138	52.0
Education		
None	26	1.2
Elementary	1338	61.1
High school	641	29.3
Vocational	10	0.5
College	162	7.4
Post-graduate	12	0.6
Occupation		
None/Students	1060	48.4
Farmers	588	26.9
Fishermen	83	3.8
Others	454	20.8
Home ownership		
No	178	8.2
Yes	2006	91.9
Land ownership		
No	818	37.5
Yes	1362	62.5
Farm ownership		
No	1357	62.3
Yes	822	37.7
Wealth status		
Wealthy	578	26.6
Medium	1326	60.9
Poor	273	12.5
MDA coverage	1760	80.4
MDA compliance in the last MDA		
Compliant	1571	71.8
Non-compliant		
Eligible but absent during MDA	406	18.5
Present but did not get treatment or consume all drugs received	192	8.8
Not classified		
Missing data	8	0.4
Eligible but were outside the province during MDA	12	0.5

In Table 2, sex, age, and occupation were found to be significantly associated with non-compliance to MDA in univariate analysis, while education and wealth status were not. Non-participation in at least one of the three previous MDA was a significant predictor of non-compliance, while mild previous infection was significantly associated with lower odds of non-compliance. Table 3 displays the univariate analysis of knowledge of schistosomiasis and MDA with non-compliance. Having heard of schistosomiasis, as well as the knowledge that it can be acquired with infected water contact, and it can be treated by taking drugs were all significantly associated with decreased probability of non-compliance. On the contrary, perceptions such as the infection can be acquired by open defecation and poor sanitation, and can be prevented by avoiding unclean drinking water increased the chance of non-adherence to MDA. Those who believe that there are other ways to treat schistosomiasis, and that there are other uses for praziquantel aside from deworming and schistosomiasis treatment were also shown to have higher risk of non-compliance. Knowledge on whether schistosomiasis can be treated and whether there are adverse reactions to taking praziquantel were also significant predictors of non-compliance.

**Table 2:** Univariate analysis of the association of demographic and socio-economic factors, history of MDA participation, and schistosomiasis infection with non-compliance to the last MDA conducted in Northern Samar, the Philippines (*n* = 2146)

Profile	Response	*N*	Non-compliant	Odds ratio	95% CI	*P*-value^a^
			*n* (%)			
Age (years)	18–30	447	158 (35.4)	1.00		
	31–40	557	134 (24.1)	0.54	0.39–0.74	<0.001
	41–50	526	106 (20.2)	0.42	0.30–0.58	<0.001
	> 50	616	194 (31.5)	0.83	0.62–1.12	0.233
Sex	Male	1029	223 (21.7)	1.00		
	Female	1117	369 (33.0)	2.04	1.64–2.59	<0.001
Education	None - Elementary	1343	356 (26.5)	1.00		
	High School	625	181 (29.0)	1.16	0.90–1.48	0.253
	Vocational, College, Post-graduate	178	55 (30.9)	1.10	0.72–1.66	0.660
Occupation	None	1038	348 (33.5)	1.00		
	Farmer	581	111 (19.1)	0.41	0.31–0.55	<0.001
	Fishing	82	25 (30.5)	0.73	0.39–1.37	0.324
	Others	445	108 (24.3)	0.49	0.36–0.68	<0.001
Wealth status	Wealthy	568	153 (26.9)	1.00		
	Medium	1309	351 (26.8)	1.04	0.79–1.38	0.770
	Poor	269	88 (32.7)	1.37	0.93–2.03	0.110
Participation in at least one of the last 3 previous MDA	No	952	392 (41.2)	1.00		
	Yes	1194	200 (16.8)	0.25	0.19–0.32	<0.001
History of schistosomiasis infection based on most recent stool survey	No infection	1552	449 (28.9)	1.00		
	Mild	517	124 (24.0)	0.71	0.54–0.93	0.014
	Moderate	55	12 (21.8)	0.58	0.27–1.24	0.160
	Severe	22	7 (31.8)	1.21	0.42–3.49	0.730
Informed about MDA activity at least one week prior to MDA	No	78	63 (80.8)	1.00		
	Yes	2068	529 (25.6)	0.05	0.03–0.11	<0.001

^a^Chi-square test

**Table 3:** Univariate analysis of the association of knowledge of schistosomiasis and MDA with non-compliance to the last MDA conducted in Northern Samar, the Philippines (*n* = 2146)

Knowledge	Response	*N*	Non-compliant	Odds ratio	95% CI	*P*-value^a^
			*n* (%)			
Has heard of schistosomiasis	No	21	14 (66.7)	1.00		
	Yes	2125	578 (27.2)	0.15	0.05–0.45	0.001
Identified at least 1 schistosomiasis sign or symptom	No	382	103 (27.0)	1.00		
	Yes	1764	489 (27.7)	0.97	0.72–1.29	0.814
How schistosomiasis is acquired						
• Contact with infected areas/waters	No	271	97 (35.8)	1.00		
	Yes	1875	495 (26.4)	0.56	0.41–0.78	<0.001
• Open defecation and poor sanitation	No	1560	398 (25.5)	1.00		
	Yes	586	194 (33.1)	1.48	1.16–1.88	0.001
• Drinking dirty water	No	1950	523 (26.8)	1.00		
	Yes	196	69 (35.2)	1.43	1.00–2.05	0.053
• Others	No	2012	563 (28.0)	1.00		
	Yes	134	29 (21.6)	0.64	0.39–1.04	0.072
Prevention and control						
• Can schistosomiasis be prevented?	No	671	168 (25.0)	1.00		
	Yes	1263	356 (28.2)	1.23	0.96–1.58	0.105
	Don’t know	212	68 (32.1)	1.40	0.94–2.08	0.094
• Avoid contact with infected water	No	1511	411 (27.2)	1.00		
	Yes	635	181 (28.5)	1.02	0.81–1.30	0.841
• Participating in MDA	No	1719	469 (27.3)	1.00		
	Yes	427	123 (28.8)	1.18	0.90–1.56	0.235
• Use sanitary toilets	No	1828	495 (27.1)	1.00		
	Yes	318	97 (30.5)	1.18	0.87–1.59	0.287
• Avoid drinking dirty water	No	1982	529 (26.7)	1.00		
	Yes	164	63 (38.4)	1.66	1.13–2.46	0.011
• Use rubber boots	No	1963	549 (28.0)	1.00		
	Yes	183	43 (23.5)	0.84	0.55–1.27	0.404
• Other prevention and control methods	No	2045	566 (27.7)	1.00		
	Yes	101	26 (25.7)	0.94	0.56–1.59	0.819
Treatment						
• Can schistosomiasis be treated?	No	119	39 (32.8)	1.00		
	Yes	1924	504 (26.2)	0.71	0.45–1.13	0.140
	Don’t know	103	49 (47.6)	1.95	1.03–3.69	0.040
• Taking drugs to treat schistosomiasis	No	729	226 (31.0)	1.00		
	Yes	1417	366 (25.8)	0.78	0.62–0.99	0.040
• Other ways schistosomiasis is treated	No	1857	492 (26.5)	1.00		
	Yes	289	100 (34.6)	1.48	1.08–2.02	0.014
• Are there adverse reactions to praziquantel?	No	577	157 (27.2)	1.00		
	Yes	1243	305 (24.5)	0.77	0.59–1.02	0.067
	Don’t know	326	130 (39.9)	2.14	1.49–3.07	<0.001
Praziquantel use						
• To treat schistosomiasis	No	1257	338 (26.9)	1.00		
	Yes	889	254 (28.6)	1.10	0.88–1.39	0.390
• For deworming	No	2072	581 (28.0)	1.00		
	Yes	74	11 (14.9)	0.49	0.24–1.01	0.054
Others	No	2077	554 (26.7)	1.00		
	Yes	69	38 (55.1)	3.12	1.76–5.54	<0.001

^a^Chi-square test

Attitudes towards schistosomiasis and MDA, for it also had significant effects on the likelihood of non-compliance based on univariate analysis, was examined (Table 4). Respondents who do not know their risk of getting schistosomiasis infection were twice as likely to be non-compliant compared to those who considered themselves of low risk. Those who do not believe in the benefits of participating in MDA had twice the risk of not adhering to the treatment than those who believe it to be beneficial. The odds of non-compliance were also higher among respondents who were unaware that the benefits of MDA were greater than the possible risk of side-effects.

**Table 4:** Univariate analysis of the association of attitudes of schistosomiasis and MDA with non-compliance to the last MDA conducted in Northern Samar, the Philippines (*n* = 2146)

Attitudes on schistosomiasis and MDA	*N*	Non-compliant	Odds ratio	95% CI	*P*-value^a^
		*n* (%)			
What do you think is your risk of becoming infected with schistosomiasis?					
No to low risk	407	88 (21.6)	1.00		
Moderate to high risk	1622	467 (28.8)	1.32	0.98–1.79	0.066
Don’t know	117	37 (31.6)	1.98	1.17–3.36	0.011
How severe do you think schistosomiasis is as a disease in your community?					
Not severe to low severity	462	112 (24.2)	1.00		
Moderate to high severity	1546	440 (28.5)	1.28	0.97–1.69	0.083
Don’t know	138	40 (29.0)	1.62	0.98–2.68	0.059
MDA is beneficial to those who participate					
Agree	1954	523 (26.8)	1.00		
Disagree	55	24 (43.6)	1.98	1.04–3.76	0.037
Don’t know	137	45 (32.9)	1.53	0.99–2.38	0.055
The benefits of MDA are greater than the possible adverse reactions					
Agree	1959	519 (26.5)	1.00		
Disagree	85	34 (40.0)	1.69	0.99–2.88	0.054
Don’t know	102	39 (38.2)	2.02	1.23–3.31	0.005

^a^Chi-square test

**Fig. 2:**
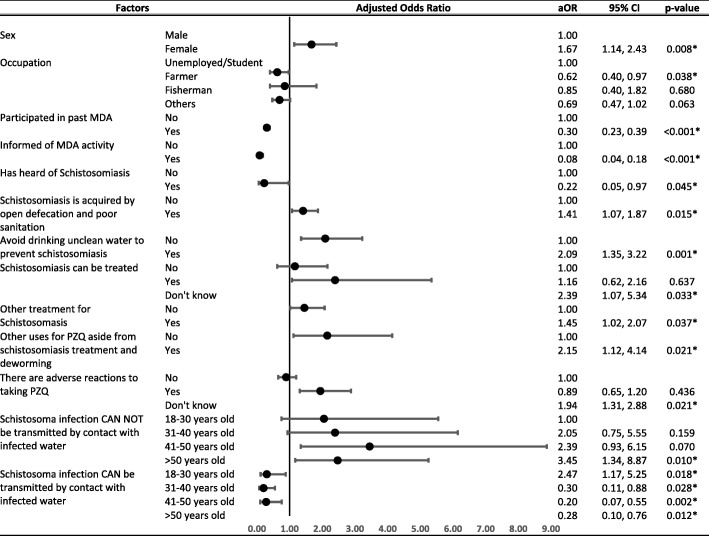
Mixed-effects logistic regression analysis of the association of knowledge of schistosomiasis and MDA, history of MDA participation, demographic and socio-economic characteristics with non-compliance to MDA among the residents of northern Samar, the Philippines (*n* = 2146). *P < 0.05

Statistically significant factors identified in the univariate analysis and interaction effects between demographic factors and knowledge were included in the stepwise backward multi-level regression analysis. The result (Fig. 2) showed that some risk factors promote non-compliance to MDA among the residents. A stepwise ‘forward’ regression was also done to check the sensitivity of the analysis. The two methods resulted in almost the same set of significant predictors, except for having heard of schistosomiasis which was only marginally significant in stepwise backward regression. Sex was a significant factor, with females (adjusted odds ratio, aOR = 1.67, *P* = 0.008) showing higher risk of non-compliance than males. Residents who believe that open defecation and poor sanitation are means of getting infected (aOR = 1.41, *P* = 0.015) and avoiding dirty drinking water can prevent schistosomiasis (aOR = 2.09, *P* = 0.001) were more likely to be non-compliant. Responses to knowledge questions of treatment were also found to have significant association with non-compliance. Those who do not know if schistosomiasis can be treated had more than twice higher risk of not adhering to MDA (aOR = 2.39, *P* = 0.033). Respondents who believe that there are other treatments for schistosomiasis aside from praziquantel (aOR = 1.45, *P* = 0.037), and that praziquantel is used for purposes other than deworming such as treatment of dengue and malaria, and schistosomiasis treatment (aOR = 2.15, *P* = 0.021) were all more likely not to take treatment.

Farmers (aOR = 0.62, *P* = 0.038) were observed as less likely to be non-compliant compared to the unemployed and students. MDA past recipients had lower odds of not complying with the recent MDA (aOR = 0.30, *P* < 0.001), while respondents who were informed at least one week prior to MDA were less likely to refuse the drug (aOR = 0.08, *P* < 0.001). Having heard of schistosomiasis was also associated with also lower risk of non-compliance to MDA (aOR = 0.22, *P* = 0.045).

Among respondents who were not aware that contact with infected water can transmit the infection, those who are over 50 years of age showed more than three times higher likelihood of non-compliance compared to the youngest age group (aOR = 3.45, *P* = 0.010). Another group who had significantly higher odds of non-compliance were the respondents of 18–30 years of age, who despite being aware that the infection can be acquired by contact with infected water, were twice more likely to be non-compliant (aOR = 2.47, *P* = 0.018). On the other hand, the older age groups 31–40 years (aOR = 0.30, *P* = 0.028), 41–50 years (aOR = 0.20, *P* = 0.002) and > 50 years (aOR = 0.28, *P* = 0.012), with knowledge on how schistosomiasis is acquired showed higher compliance to MDA.

## Discussion

As of 2016, fifteen years after preventive chemotherapy had been endorsed *via* WHA 54.19 as the main strategy for control of schistosomiasis, the global reported coverage rate is 35.6% [3]. With slow progress in improving coverage, the target of attaining a minimum coverage of 75% among school-aged children by 2020 may be an impossible feat. Issues of drug availability are being resolved with increased donations but measures should also be taken to increase drug coverage rates and address factors responsible for non-compliance [3].

The coverage in the present study is almost twice higher than the 43.5% WHO reported coverage in the country in 2015 [13]. The elevated reported coverage in this study may be explained by the fact that the study area has been part of an integrated control trial since 2012 [23]. The enhanced MDA coverage may also be due to additional funding for provision of snacks to participants when taking the treatment and incentives to health workers and volunteers from the project. Pre-treatment snacks, which are given to prevent treatment side effects, has been shown to improve coverage in Uganda [26]. A review on schistosomiasis MDA programs has identified lack of financial or material incentives as causes of low morale and performance among community drug distributors [27].

The present study observed a gap of 9% between the MDA coverage (81%) and compliance (72%). A similar coverage-compliance gap was observed in MDA for schistosomiasis in Zanzibar [17]. Hence, despite the moderately high coverage rate found here, nationwide compliance remains low. Given the existence of gaps in coverage rates and patient compliance, the proportion of the population who are actually treated may be too small to make significant strides towards disease elimination [13, 14]. This is compounded by the zoonotic nature of the disease in the Philippines were bovines and dogs act as significant reservoir hosts [28, 29].

A study of factors related to MDA drug coverage for schistosomiasis has shown that males are less likely to receive treatment, while other studies found that sex is not a significant determinant of compliance [15, 30–32]. In our study, females showed a higher risk of non-compliance, similar to the results found in Western Samar, the Philippines [22]. Among women who did not comply with treatment, 139 (38%) cited breastfeeding as the main reason for not receiving treatment while 23 (6%) said that they were pregnant at the time of MDA. This is despite the inclusion of lactating mothers and pregnant women in the eligible population in WHO’s manual for preventive chemotherapy [5]. The DOH guideline for treatment also presents evidence of the benefits of giving praziquantel to pregnant and lactating women that are diagnosed with schistosomiasis, but it was not clearly specified if they can be given the drug empirically during MDA campaigns. In a study in Nigeria, as high as 21% of pregnant women were positive for *S. haematobium,* indicating that they can be a significant source of infection that may lead to continued transmission if this population is left untreated [33]. Infected pregnant women, just like the non-pregnant population, are also at risk of developing morbidities such as undernutrition, anemia, hepatic fibrosis and esophageal varices [34]. Despite WHO’s recommendation in 2006, countries like Zanzibar, Tanzania, China, Gabon, Uganda and Kenya have yet to adopt policies on treating infected pregnant women with praziquantel [34].

In this study the reason for non-compliance for the majority of respondents who did not participate in the previous and present MDA were due to the following: not being at home (*n* = 125, 32%) at the time of treatment; did not know about the MDA activity (*n* = 58, 15%); fear of treatment side effects (*n* = 47, 12%); not infected, hence, do not feel the need for treatment (*n* = 39, 10%); and a general dislike for taking medication (*n* = 32, 8%). The poor compliance among those who were unaware of the MDA activity suggests the need for proper and effective notice before mass treatment at the village level. Eighty percent of those who were informed said that they first learned of the activity within a week from the date of MDA, while almost a tenth heard about it on the day itself. The majority of the people received notice from the public address system of the barangay, a megaphone carried around by an official of the community who is making the announcement. This is often done during the day, when a lot of the people are at work or in school. The announcements might not reach those who live away from the center of the community, especially in outlying areas that are difficult to access.

The prevalence of *Schistosoma* infection is very high among bovines which are used in farming in the province thus putting farmers at high risk [28]. Their risk is indicated by the high prevalence of infection among farmers (41.5%), the second highest by occupation, in a 2012 survey [25]. Hence, it was not surprising that they showed better compliance to MDA than those who were unemployed or students. In contrast, a study in Uganda has shown higher intensity of infection was significantly associated with decreased odds of compliance to treatment [18]. In this study, infection status had no significant association with non-compliance in the multivariate analysis. However, univariate analysis showed that mild *Schistosoma* infection is significantly associated with better compliance than those who had no infection. This may be due to the fact that drug distributors were aware of who among the population were positive during the stool survey, and tried to locate them during MDA to ensure that they received treatment.

Studies have shown that knowledge of schistosomiasis is associated with increased likelihood of uptake of praziquantel during MDA [15, 31]. This is supported by the findings of this study, were respondents who have heard of schistosomiasis were more likely to comply with treatment. Those who were aware that schistosomiasis can be transmitted by contact with infected waters were also less likely to be non-compliant to treatment. Moreover, the study also showed that lack of knowledge and the presence of misconceptions about schistosomiasis treatment and prevention can result to higher likelihood of non-compliance. Uncertainties about whether schistosomiasis can be treated and that treatment can result in adverse reactions has led many respondents against taking the drug. The misconception that *Schistosoma* infection can be acquired by drinking or gargling unclean or contaminated water was found to be very common among researchers exploring knowledge of schistosomiasis [35, 36]. The feeling that they do not need treatment was often cited as reason for non-compliance in MDA [16, 37, 38]. The same is true for those who think that there are other ways to treat schistosomiasis such as other medications, using traditional medicine, vaccination, or taking vitamin supplements. Some of the respondents thought praziquantel was for treating other diseases such as dengue or malaria. In Tanzania the misunderstanding that the drugs being used to treat filariasis were for contraception or an inhibitor for sexual drive resulted in a lower drug coverage rate [11].

## Conclusions

This study has identified that those more likely to be MDA non-compliant were females, young adults, uninformed residents, and those non-compliant to previous MDA campaigns (Table 5). The administration of praziquantel to pregnant and lactating women, based on WHO recommendations and a recent study published on the safety of praziquantel for pregnant women in the Philippines, needs to be clearly translated into national policy [39]. Strategies to inform residents of pending MDA at the village level should ensure that everyone will have the opportunity to be treated, including those who are studying, working, or living hard to reach areas. There is an urgent need to identify systematic non-compliers in the population so that they can be targeted for health education. This study has shown that lack of knowledge and the presence of misconceptions about disease transmission, prevention and treatment significantly increases non-compliance. Addressing misconceptions through health education will ultimately increase drug compliance at the barangay level [40]. The global control of schistosomiasis is at a critical stage. Drug coverage rates must be improved and we must be certain that populations at risk comply with free treatment through directly observed therapy. Health-care providers of MDA must be knowledgeable of the disease in order to advise and inform residents at the local level. If achieved disease elimination may become a reality in our lifetime.

**Table 5:** Profile of MDA study non-compliance and recommendations to increase treatment uptake

Profile of MDA non-compliant	Recommendations
Female	• Clearly define and list down the inclusion and exclusion criteria for MDA, based on WHO recommendations and recent published studies on the safety of praziquantel for pregnant women. Pregnant and lactating women should be included in the eligible population for MDA
	• This information should be given to those in the frontline of MDA implementation like the doctors, nurses, and community volunteers, as well as to the people in the community
Younger (18–30) age group	• Schedule MDA activities at a time that would cause the least disturbance in the work or school activities of the students
Non-compliant in previous MDA	• Should be targeted for intensive health education
Uninformed about MDA activities	• Inform the community about MDA schedule at least a week before the treatment day
	• In addition to the use of the public address system, the Barangay (Village) Health Workers and/or Officials should visit each household to inform them of the MDA schedule.
Has not heard of schistosomiasis	• A health education program that is designed not only to give out information about schistosomiasis and MDA, but also to correct the misconceptions that are prevailing in the population
Has no knowledge or misconceptions about:	
○ disease transmission	
○ disease prevention	
○ disease treatment	
